# Supplementary material for: Toxoplasmosis accelerates the progression of hereditary spastic paraplegia
Source: mSphere. 2025 Mar 18;10(4):e00826-24. doi: 10.1128/msphere.00826-24 (PMC12039240; doi:10.1128/msphere.00826-24)
Supplement: Fig. S1 — Infected animals exhibit minimal brain cyst presence. [file msphere.00826-24-s0001.pdf]

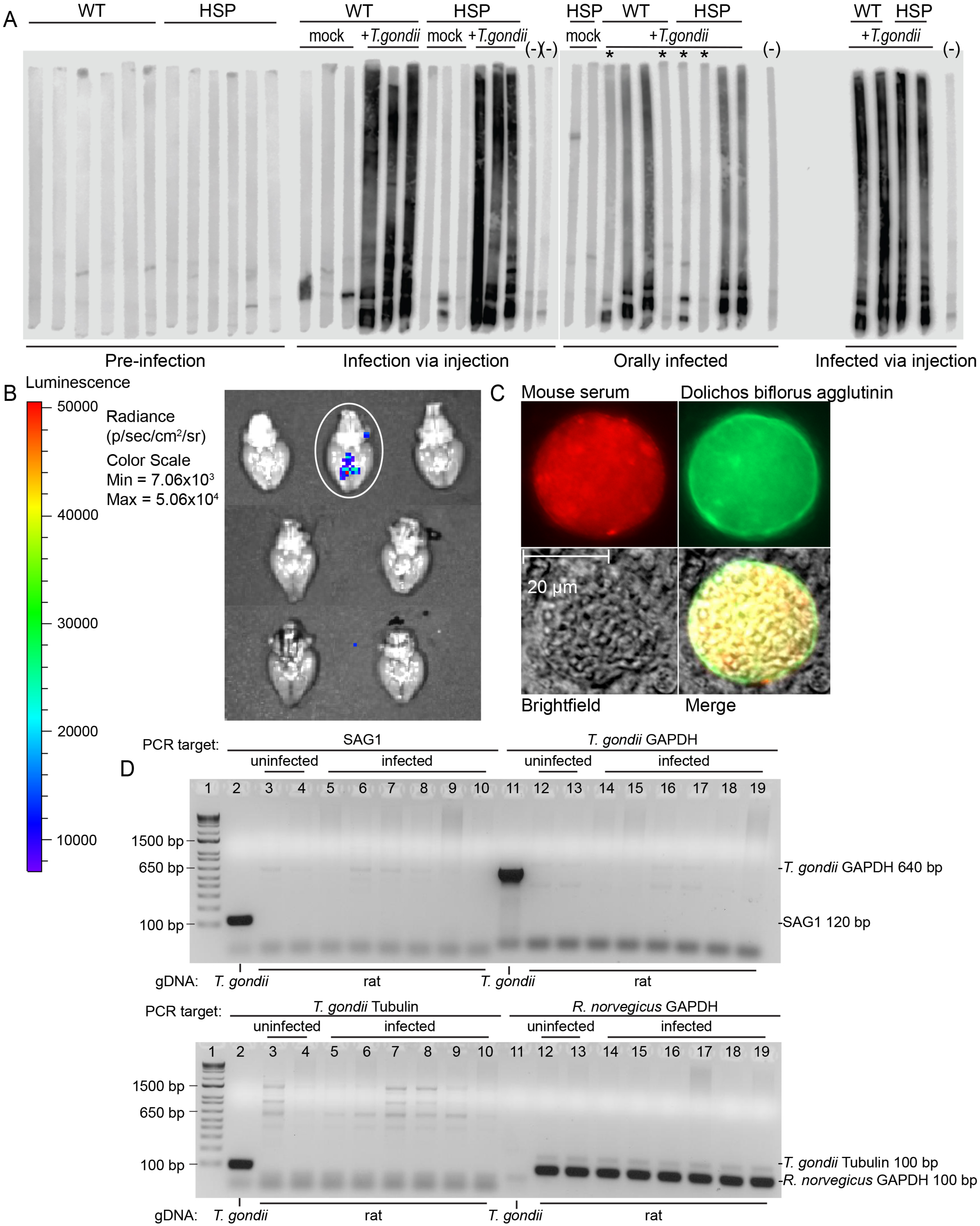
A
WT
HSP
WT
HSP
mock
+T.gondii
mock
+T.gondii
(-)
(-)
HSP
WT
HSP
mock
+T.gondii
(-)
WT
HSP
+T.gondii
(-)
Pre-infection
Infection via injection
Orally infected
Infected via injection
B
Luminescence
50000
40000
30000
20000
10000
Radiance
(p/sec/cm²/sr)
Color Scale
Min = 7.06x10³
Max = 5.06x10⁴
C
Mouse serum
Dolichos biflorus agglutinin
20 µm
Brightfield
Merge
D
PCR target:
SAG1
T. gondii GAPDH
uninfected
infected
1 2 3 4 5 6 7 8 9 10 11 12 13 14 15 16 17 18 19
1500 bp
650 bp
100 bp
T. gondii GAPDH 640 bp
SAG1 120 bp
gDNA:
T. gondii
rat
PCR target:
T. gondii Tubulin
R. norvegicus GAPDH
T. gondii Tubulin 100 bp
R. norvegicus GAPDH 100 bp

Figure S1. Infected animals exhibit minimal brain cyst presence. (A) Sera was taken from rats at experiment termination at 7 weeks post-infection. Western blot strips from *T. gondii* antibody test. *T. gondii* proteins were separated via SDS-PAGE, transferred to nitrocellulose, incubated with the indicated rat sera at a 1/1000 dilution, and exposed to anti-rat HRP. (-) = secondary antibody only. Strips marked with * denote animals deemed incompletely infected and excluded from further analysis. (B) Representative *in vivo* imaging system (IVIS) results of infected brains of Sprague-Dawley rats following treatment with 10 mg/mL luciferin. The five brains on the upper two rows were harvested from animals injected with 10^7 tachyzoites, while the two brains on the lower row were from animals injected with 10^8 tachyzoites. Radiance scale on left. (C) Representative images of a *T. gondii* cyst harvested from the circled brain in Fig. S1B. Brain tissue was homogenized and stained using serum from a mouse with a chronic *T. gondii* infection (*top left*, red) and *Dolichos biflorus* agglutinin (*top right*, green). Brightfield images of the cyst and encased parasites (*bottom left*, gray) and merge (*bottom right*). Scale bar, 20 µm. (D) PCR of genomic DNA (gDNA). Lanes 3, 5, 7, 12, and 14-16 are wild-type rat gDNA; lanes 4, 8-10, 13, and 17-19 are hereditary spastic paraplegia mutant rat gDNA. Glyceraldehyde-3-phosphate dehydrogenase (GAPDH), base pairs (bp).
