# Supplementary material for: Toxoplasmosis accelerates the progression of hereditary spastic paraplegia
Source: mSphere. 2025 Mar 18;10(4):e00826-24. doi: 10.1128/msphere.00826-24 (PMC12039240; doi:10.1128/msphere.00826-24)
Supplement: Fig. S2 — Seronegative infected rats have less severe neurological disease than seropositive rats. [file msphere.00826-24-s0002.pdf]

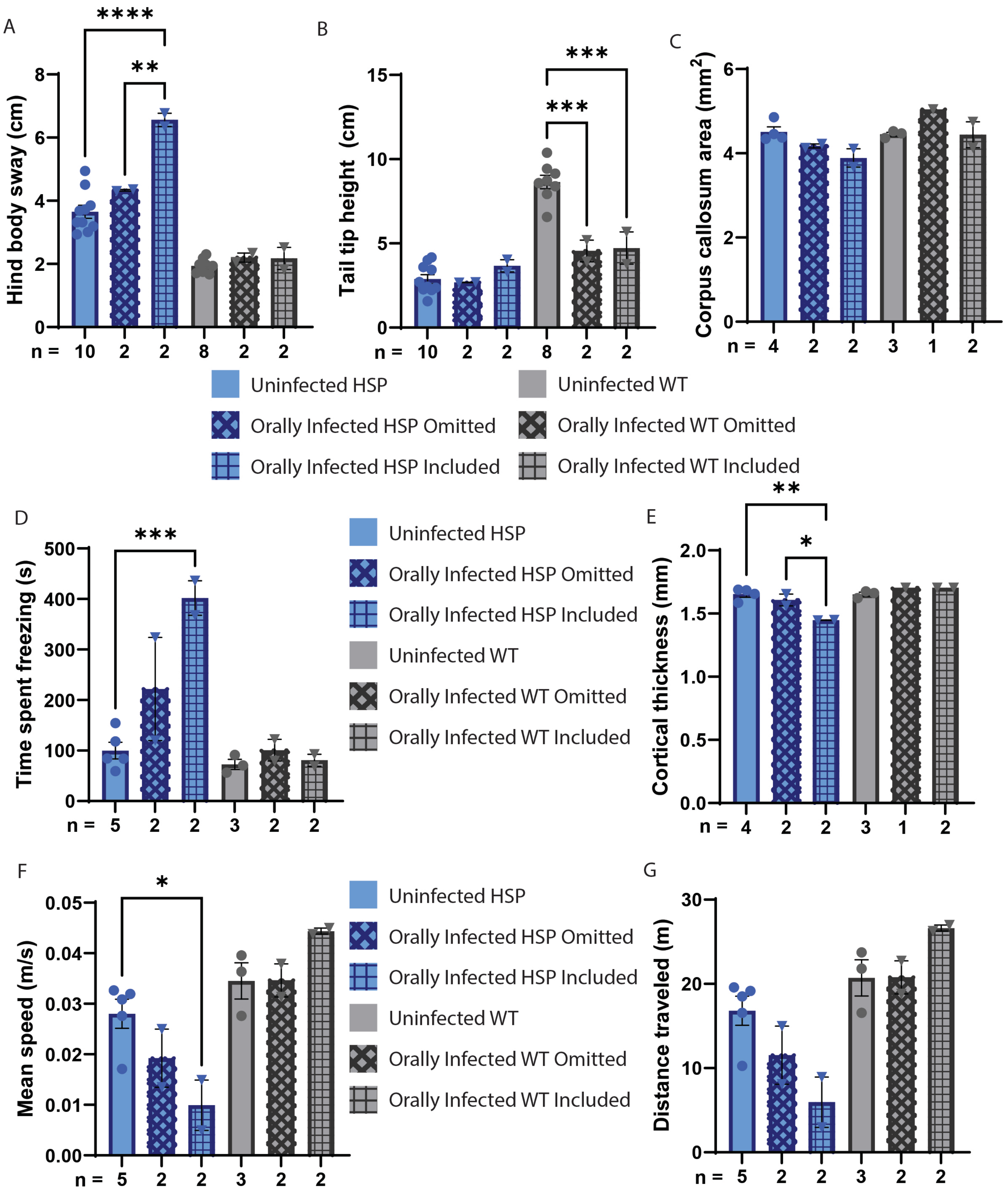
A
Hind body sway (cm)
****
**
n = 10 2 2 8 2 2
B
Tail tip height (cm)
***
***
n = 10 2 2 8 2 2
C
Corpus callosum area (mm²)
n = 4 2 2 3 1 2
Uninfected HSP
Orally Infected HSP Omitted
Orally Infected HSP Included
Uninfected WT
Orally Infected WT Omitted
Orally Infected WT Included
D
Time spent freezing (s)
***
n = 5 2 2 3 2 2
E
Cortical thickness (mm)
**
*
n = 4 2 2 3 1 2
F
Mean speed (m/s)
*
n = 5 2 2 3 2 2
G
Distance traveled (m)
n = 5 2 2 3 2 2

Fig. S2. Seronegative infected rats have less severe neurological disease than seropositive rats. Measurements of hind body sway (A), tail tip height (B), corpus callosum area (C), time spent freezing (D), cortical thickness (E), average speed (F), and distance traveled (G) of animals of the indicated genotype and infection condition. Data points represent average measurements from individual animals. Triangle symbols represent data points from animals infected via injection, while inverted triangle symbols represent data from animals infected orally. Error bars represent mean ± SEM. $**P < 0.01$, $***P < 0.001$, and $****P < 0.0001$, as calculated using Tukey's multiple comparisons test. wt, wild-type; HSP, hereditary spastic paraplegia mutant.
