# Supplementary material for: Toxoplasmosis accelerates the progression of hereditary spastic paraplegia
Source: mSphere. 2025 Mar 18;10(4):e00826-24. doi: 10.1128/msphere.00826-24 (PMC12039240; doi:10.1128/msphere.00826-24)
Supplement: Fig. S3 — Orally infected animals exhibit more severe symptoms than animals infected via intraperitoneal injection. [file msphere.00826-24-s0003.pdf]

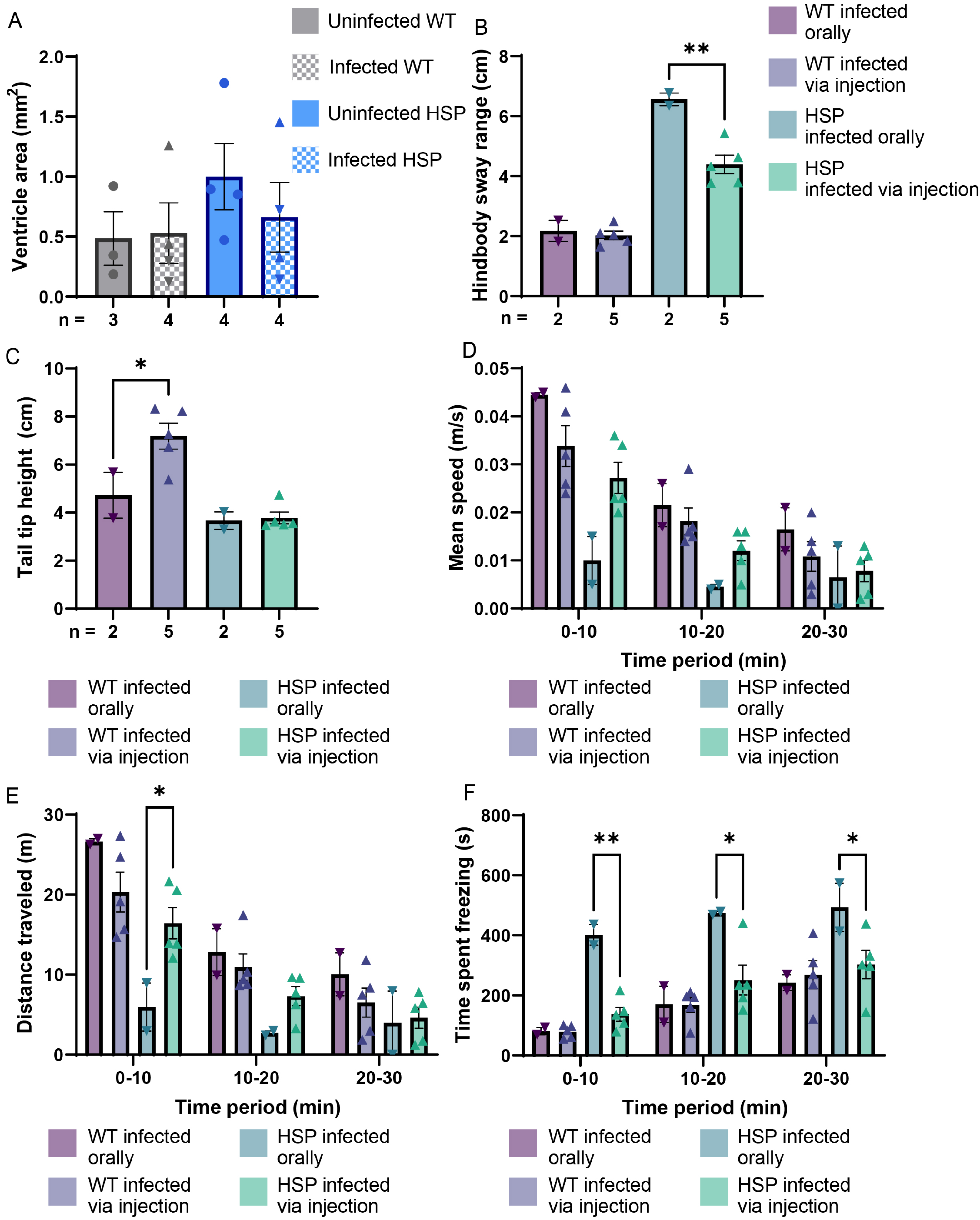
A
B
C
D
E
F
Ventricle area (mm²)
Uninfected WT
Infected WT
Uninfected HSP
Infected HSP
n = 3 4 4 4
Hindbody sway range (cm)
n = 2 5 2 5
Tail tip height (cm)
Mean speed (m/s)
Distance traveled (m)
Time spent freezing (s)
Time period (min)
0-10
10-20
20-30
WT infected orally
WT infected via injection
HSP infected orally
HSP infected via injection

Fig. S3. Orally infected animals exhibit more severe symptoms than animals infected via intraperitoneal injection. (A) Ventricle area was not altered in infected animals. (B,C) Gait measurements of hind body sway (B) and tail tip height (C) of animals of the indicated genotype and infection method. Data points represent measurements from individual animals. Error bars represent mean ± SEM. *$P < 0.05$ and **$P < 0.01$, as calculated using Tukey's multiple comparisons test. wt, wild-type. (D-F) Measurements of distance traveled (D), average speed (E), and time spent freezing (F) in the open field test by animals of the indicated genotype and infection condition. Data points represent measurements from individual animals. Orally infected wild-type, n = 2; intraperitoneally (IP) infected wild-type, n = 5; orally infected HSP mutant, n = 2; IP infected HSP mutant, n = 5. Error bars represent mean ± SEM. Comparisons between all groups were performed but only the comparisons reaching significance were marked. *$P < 0.05$ and **$P < 0.01$, as calculated using Tukey's multiple comparisons test. wt, wild-type. HSP, hereditary spastic paraplegia mutant.
