# Supplementary material for: Toxoplasmosis accelerates the progression of hereditary spastic paraplegia
Source: mSphere. 2025 Mar 18;10(4):e00826-24. doi: 10.1128/msphere.00826-24 (PMC12039240; doi:10.1128/msphere.00826-24)
Supplement: Fig. S4 — Brain morphology did not differ between infection conditions; T. gondii infection did not cause gliosis. [file msphere.00826-24-s0004.pdf]

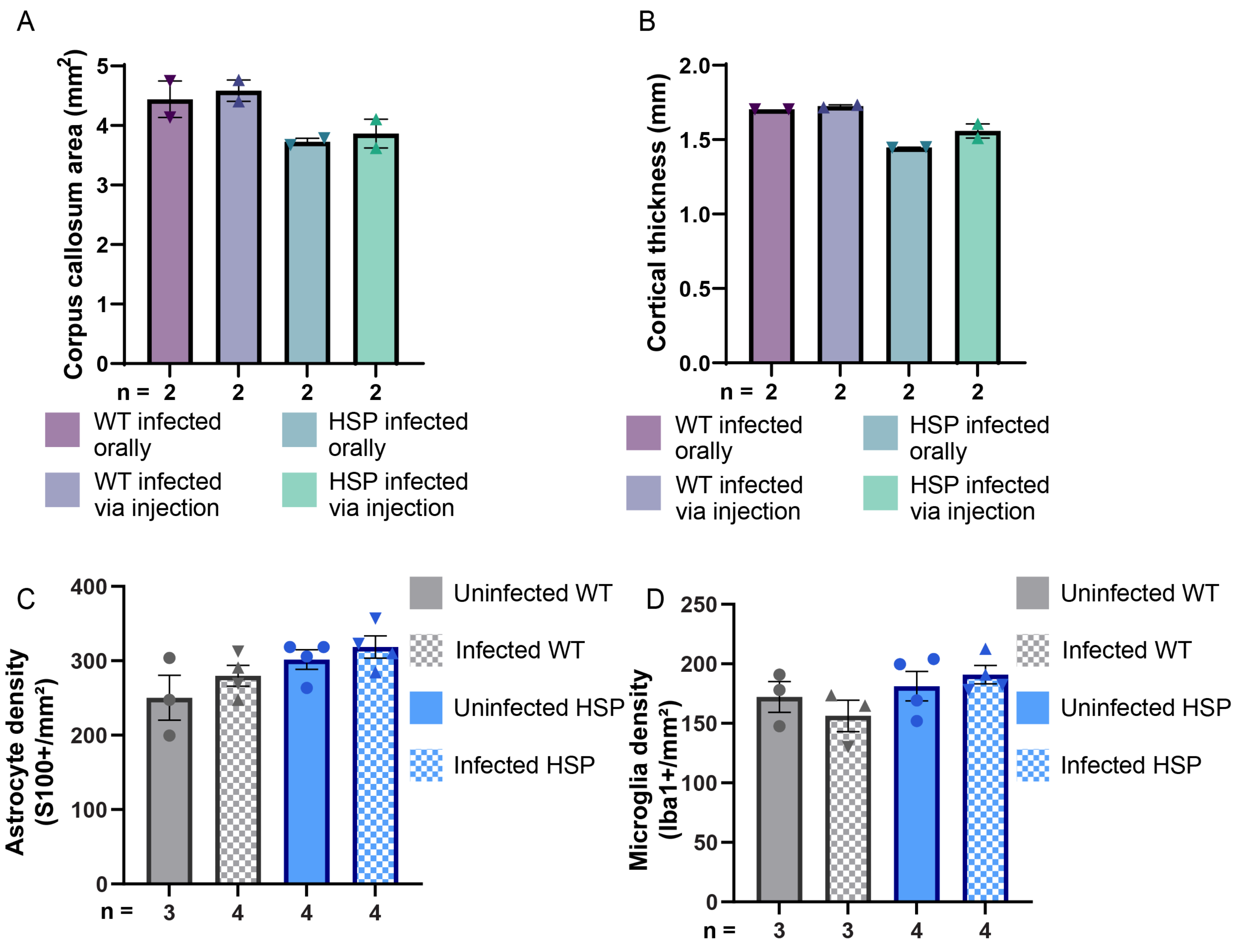

A
Corpus callosum area (mm²)
0
1
2
3
4
5
n = 2
2
2
2
WT infected orally
WT infected via injection
HSP infected orally
HSP infected via injection
B
Cortical thickness (mm)
0.0
0.5
1.0
1.5
2.0
n = 2
2
2
2
WT infected orally
WT infected via injection
HSP infected orally
HSP infected via injection
C
Astrocyte density (S100+/mm²)
0
100
200
300
400
n = 3
4
4
4
Uninfected WT
Infected WT
Uninfected HSP
Infected HSP
D
Microglia density (Iba1+/mm²)
0
50
100
150
200
250
n = 3
3
4
4
Uninfected WT
Infected WT
Uninfected HSP
Infected HSP

Fig. S4. Brain morphology did not differ between infection conditions; *T. gondii* infection did not cause gliosis. Measurements of corpus callosum area (A) and cortical thickness (B) of animals of the indicated genotype and infection method. Data points represent measurements from individual animals. Error bars represent mean ± SEM. (C-D) Measurements of astrocyte (C) and microglia (D) density in brains of animals of the indicated genotype and infection condition. Data points represent measurements from individual animals. Error bars represent mean ± SEM.
