# Supplementary material for: Toxoplasmosis accelerates the progression of hereditary spastic paraplegia
Source: mSphere. 2025 Mar 18;10(4):e00826-24. doi: 10.1128/msphere.00826-24 (PMC12039240; doi:10.1128/msphere.00826-24)
Supplement: Fig. S5 — PCA analysis shows one infected HSP mutant sample was an outlier. [file msphere.00826-24-s0005.pdf]

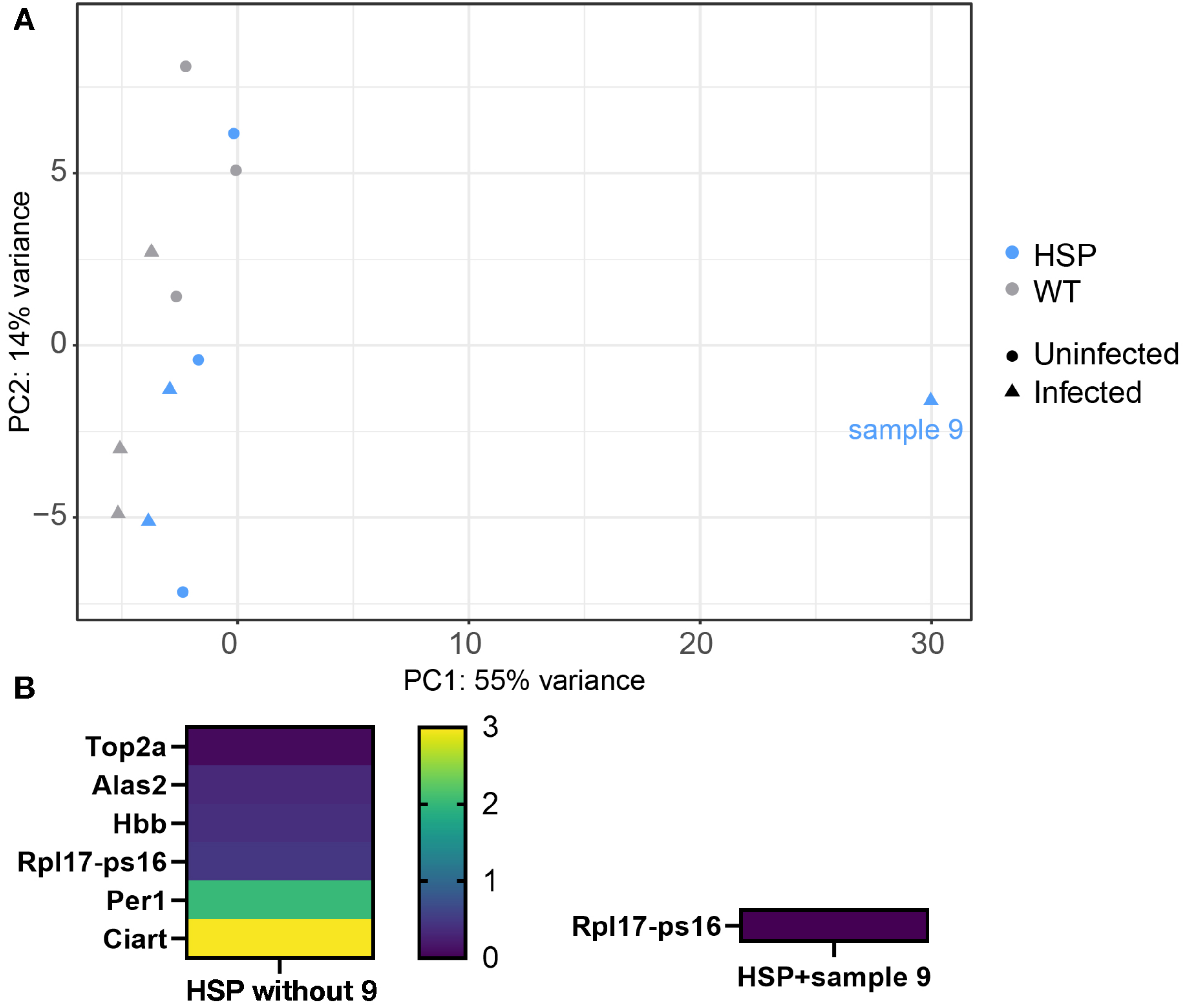
A
PC2: 14% variance
PC1: 55% variance
5
0
−5
0
10
20
30
sample 9
HSP
WT
Uninfected
Infected
B
Top2a
Alas2
Hbb
Rpl17-ps16
Per1
Ciart
HSP without 9
3
2
1
0
Rpl17-ps16
HSP+sample 9

Fig. S5. PCA analysis shows one infected HSP mutant sample was an outlier. (A) PCA plot of RNA sequencing data. Brain tissue from the motor cortex was snap frozen in TRIzol and RNA was isolated. High-quality RNA was submitted for RNA sequencing. Based on PCA analysis, sample 9 was an outlier and excluded from further analysis. PCA, principal component analysis. (B) Heatmaps comparing HSP transcript abundance, without (left) and with (right) sample 9.
