# Supplementary material for: Toxoplasmosis accelerates the progression of hereditary spastic paraplegia
Source: mSphere. 2025 Mar 18;10(4):e00826-24. doi: 10.1128/msphere.00826-24 (PMC12039240; doi:10.1128/msphere.00826-24)
Supplement: Fig. S6 — Gene ontology classification of genes differentially regulated upon T. gondii infection in wild-type animals. [file msphere.00826-24-s0006.pdf]

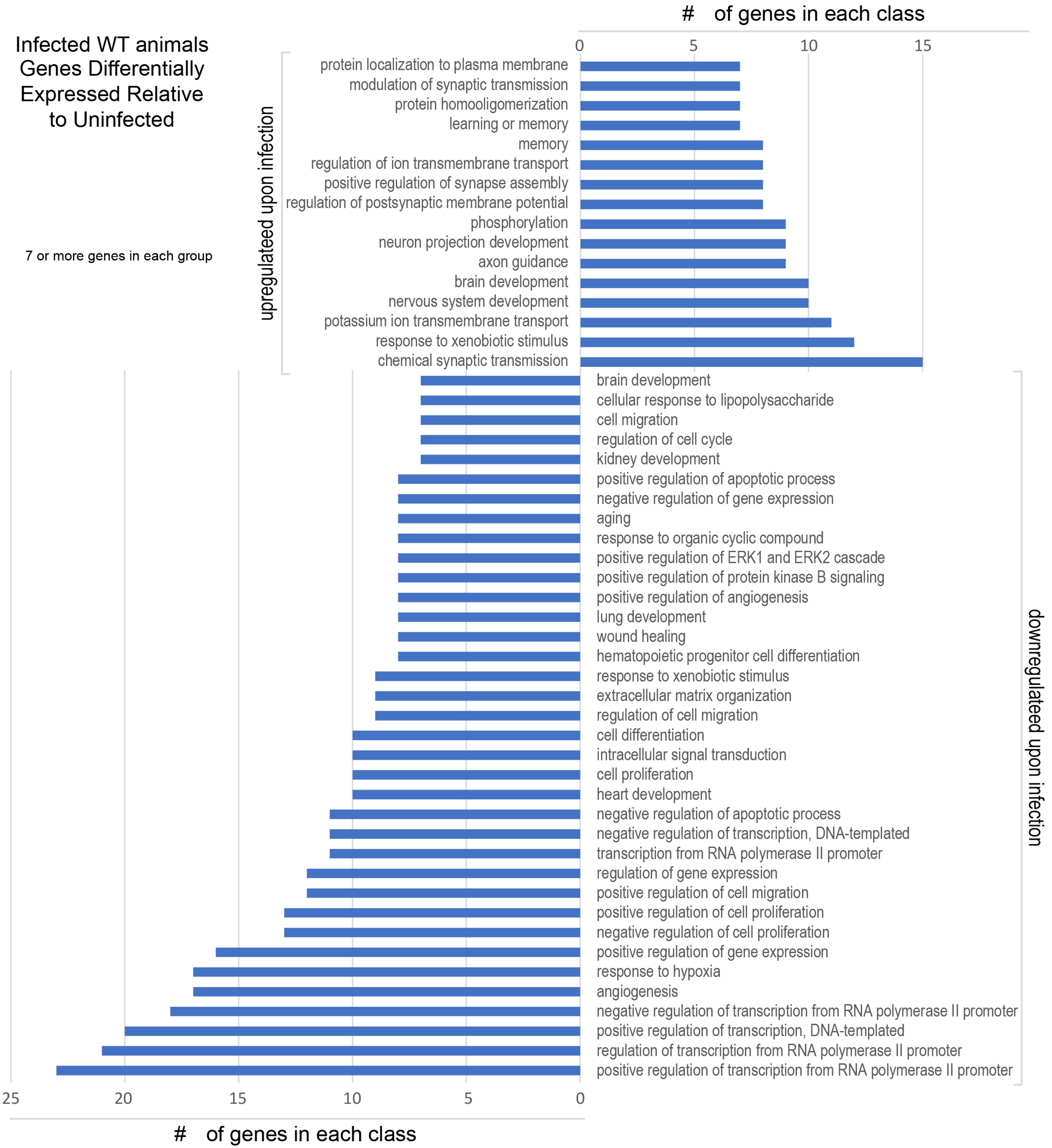
Infected WT animals Genes Differentially Expressed Relative to Uninfected
7 or more genes in each group
# of genes in each class
0
5
10
15
upregulated upon infection
protein localization to plasma membrane
modulation of synaptic transmission
protein homooligomerization
learning or memory
memory
regulation of ion transmembrane transport
positive regulation of synapse assembly
regulation of postsynaptic membrane potential
phosphorylation
neuron projection development
axon guidance
brain development
nervous system development
potassium ion transmembrane transport
response to xenobiotic stimulus
chemical synaptic transmission
downregulated upon infection
brain development
cellular response to lipopolysaccharide
cell migration
regulation of cell cycle
kidney development
positive regulation of apoptotic process
negative regulation of gene expression
aging
response to organic cyclic compound
positive regulation of ERK1 and ERK2 cascade
positive regulation of protein kinase B signaling
positive regulation of angiogenesis
lung development
wound healing
hematopoietic progenitor cell differentiation
response to xenobiotic stimulus
extracellular matrix organization
regulation of cell migration
cell differentiation
intracellular signal transduction
cell proliferation
heart development
negative regulation of apoptotic process
negative regulation of transcription, DNA-templated
transcription from RNA polymerase II promoter
regulation of gene expression
positive regulation of cell migration
positive regulation of cell proliferation
negative regulation of cell proliferation
positive regulation of gene expression
response to hypoxia
angiogenesis
negative regulation of transcription from RNA polymerase II promoter
positive regulation of transcription, DNA-templated
regulation of transcription from RNA polymerase II promoter
positive regulation of transcription from RNA polymerase II promoter
25
20
15
10
5
0
# of genes in each class

Fig. S6. Gene ontology classification of genes differentially regulated upon *T. gondii* infection in wild-type animals. Database for Annotation, Visualization and Integrated Discovery (DAVID) gene ontology tools were used to classify genes differentially expressed in the primary motor region of wild type animals. Genes whose expression differed at least 30% upon infection were included in the analysis. Groups containing at least 7 differentially expressed genes are shown. wt, wild-type.
