# Supplementary material for: Toxoplasmosis accelerates the progression of hereditary spastic paraplegia
Source: mSphere. 2025 Mar 18;10(4):e00826-24. doi: 10.1128/msphere.00826-24 (PMC12039240; doi:10.1128/msphere.00826-24)
Supplement: Legends — Table S1 and Movie S1 legends. [file msphere.00826-24-s0007.docx]

**Table S1. List of genes differentially expressed in the primary motor region of wild-type animals upon chronic *T. gondii* infection.** Genes whose expression differed at least 30% upon infection are shown. RGD, Rat Genome Database.

**Movie S1. Representative videos of 13-week male rats crossing a transparent platform.** A camera underneath the platform uses mirrors to capture three views of rat motion. Colored points added by analysis software. Uninfected wild-type (*top left*), uninfected HSP mutant (*top right*), infected wild-type (*bottom left*), and infected HSP mutant (*bottom right*) animals are shown. Playback speed is 0.5x real time.
